# Supplementary material for: Identification of Single Yeast Budding Using Impedance Cytometry with a Narrow Electrode Span
Source: Sensors (Basel). 2022 Oct 12;22(20):7743. doi: 10.3390/s22207743 (PMC9609181; doi:10.3390/s22207743)
Supplement: Supplementary file 1 [file sensors-22-07743-s001.zip › sensors-1913950-supplementary.pdf]

Supporting information

Identification of Single Yeast Budding Using Impedance Cytometry with a Narrow Electrode Span

Xun Liu [1], Tao Tang [1,*], Po-Wei Yi [1,2], Yapeng Yuan [3], Cheng Lei [4], Ming Li [5], Yo Tanaka [3], Yoichiroh Hosokawa [1] and Yaxiaer Yalikun [1,*]

[1] Division of Materials Science, Nara Institute of Science and Technology, 8916-5 Takayama-cho, Ikoma 630-0192, Nara, Japan

[2] Department of Applied Chemistry, National Yang Ming Chiao Tung University, Hsinchu 30010, Taiwan

[3] Center for Biosystems Dynamics Research (BDR), RIKEN, 1-3 Yamadaoka, Suita 565-0871, Osaka, Japan

[4] The Institute of Technological Sciences, Wuhan University, Wuhan 430072, China

[5] School of Engineering, Macquarie University, Sydney 2109, Australia

* Correspondence: tang.tao.ts3@ms.naist.jp (T.T.); yaxiaer@ms.naist.jp (Y.Y.)

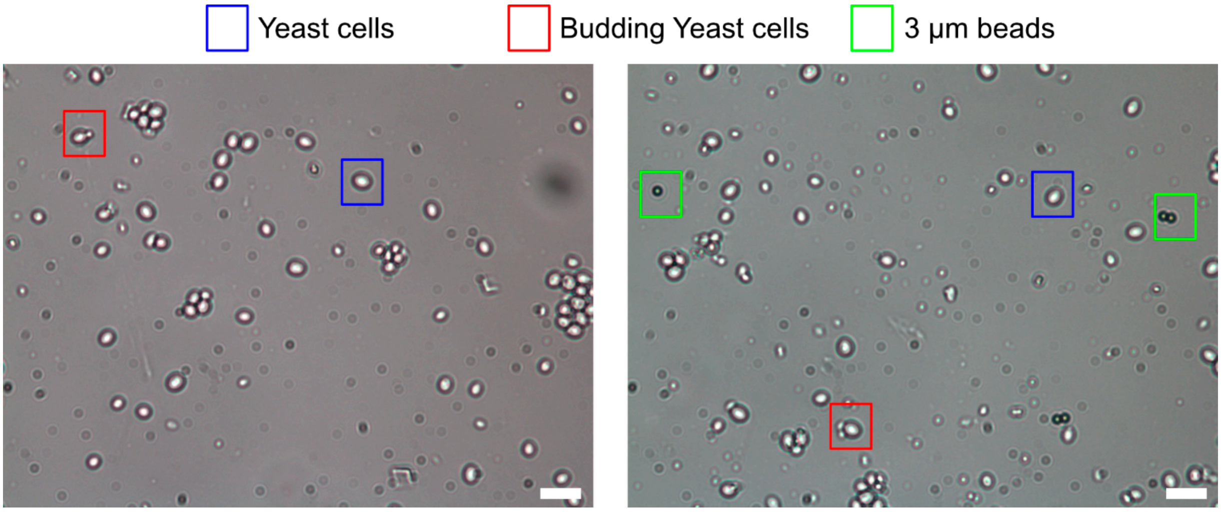

Figure S1. The photos of yeast sample in experiments, three kinds of objects are marked in pictures; the number of budding yeasts is lower than normal yeast cells. In the two photos, there are about 7 budding cells and more than 80 yeast cells, the ratio is about 1:10.
